# Gamma-Ray and Radio-Frequency Radiation from Thunderstorms Observed from Space and Ground

**DOI:** 10.1038/s41598-020-63437-2

**Published:** 2020-04-29

**Authors:** B. G. Mailyan, A. Nag, J. R. Dwyer, R. K. Said, M. S. Briggs, O. J. Roberts, M. Stanbro, H. K. Rassoul

**Affiliations:** 1 Florida Institute of Technology, Melbourne, Florida USA; 2 The University of Alabama in Huntsville, Huntsville, Alabama USA; 3 University of New Hampshire, Durham, New Hampshire USA; 4 Vaisala Inc., Louisville, Colorado, USA; 5 Universities Space Research Association, Huntsville, Alabama USA

**Keywords:** Atmospheric science, Space physics

## Abstract

Terrestrial gamma ray flashes (TGFs) are a class of enigmatic electrical discharges in the Earth’s atmosphere. In this study, we analyze an unprecedentedly large dataset comprised of 2188 TGFs whose signatures were simultaneously measured using space- and ground-based detectors over a five-year period. The Gamma-ray Burst Monitor (GBM) on board the Fermi spacecraft provided the energetic radiation measurements. Radio frequency (RF) measurements were obtained from the Global Lightning Dataset (GLD360). Here we show the existence of two categories of TGFs − those that were accompanied by quasi-simultaneous electromagnetic pulses (EMPs) detected by the GLD360 and those without such simultaneous EMPs. We examined, for the first time, the dependence of the TGF-associated EMP-peak-amplitude on the horizontal offset distance between the Fermi spacecraft and the TGF source. TGFs detected by the GBM with sources at farther horizontal distances are expected to be intrinsically brighter and were found to be associated with EMPs having larger median peak-amplitudes. This provides independent evidence that the EMPs and TGFs are produced by the same phenomenon, rather than the EMPs being from “regular” lightning in TGF-producing thunderstorms.

## Introduction

Terrestrial gamma ray flashes or TGFs, a phenomenon discovered relatively recently by Fishman *et al*.^1^, are brief (typically less than 1 ms) bursts of energetic gamma-ray photons generated during thunderstorms. TGFs are one of the highest-energy (10–20 MeV or more) natural photon emissions on Earth. The mechanism for creating the causative high-energy electrons in the source region still remains unclear. Some models assume that charged particles are accelerated in the relatively-high electric field region concentrated at the tips of lightning leaders^2,3^, while other models consider the particle-acceleration to occur in large-scale electric fields in thunderclouds^4,5^. A leading hypothesis for TGF-production is the Relativistic Runaway Electron Avalanche (RREA)^6,7^ process followed by bremsstrahlung photon emission occurring in the presence of thundercloud electric fields. However, some important parameters of the processes leading to the production of TGFs, such as the spatial-scale of the electron-acceleration region and the source of the seed particles necessary for the RREA processes, as well as the current and charge-transfer characteristics of TGFs remain unknown. TGFs are typically detected worldwide by satellite-based instruments^1,8^, but several ground-based^9–14^ and two aircraft-based^15,16^ observations have been made. TGFs reported from ground-based observations are associated with (usually high-intensity) cloud-to-ground discharges effectively transporting negative charge to ground. The fact that TGFs are often accompanied by radio-frequency electromagnetic pulses (EMPs) was not known until very recently^17^. These accompanying relatively-high-amplitude EMPs have been measured by sensors operating in the low and very low frequency (VLF) ranges^18,19^. These EMPs are also geolocated by ground-based lightning locating systems (LLSs)^20–26^. Connaughton *et al*.^21^ found that the rate of association between Fermi GBM-TGFs and LLS-reported VLF EMPs depended strongly upon TGF durations and they interpreted EMPs that occurred almost simultaneously with TGF photon-count peak-times to be the VLF signatures of relativistic electrons and their resulting ionization. However, further evidence is needed to determine whether these EMPs are signatures of lightning that “triggers” the TGFs or whether they are RF signatures of the TGFs themselves. Also, not all TGFs are accompanied by EMPs that are detected by LLS. Note that LLSs are tuned to report EMPs from lightning having durations of about a microsecond to several hundred microseconds and estimated source-peak-currents of few kiloamperes or more^27^. The question remains as to why some TGFs are not accompanied by LLS-detectable EMPs. This could be related to the source-intensity, discharge-duration, frequencies of emission, radiation pattern (orientation), or location of the source region within thunderclouds of such TGFs being significantly different than TGFs whose accompanying-EMPs are reported by LLSs.

### TGFs with and without LLS-detectable simultaneous EMPs

**Figure 1:**
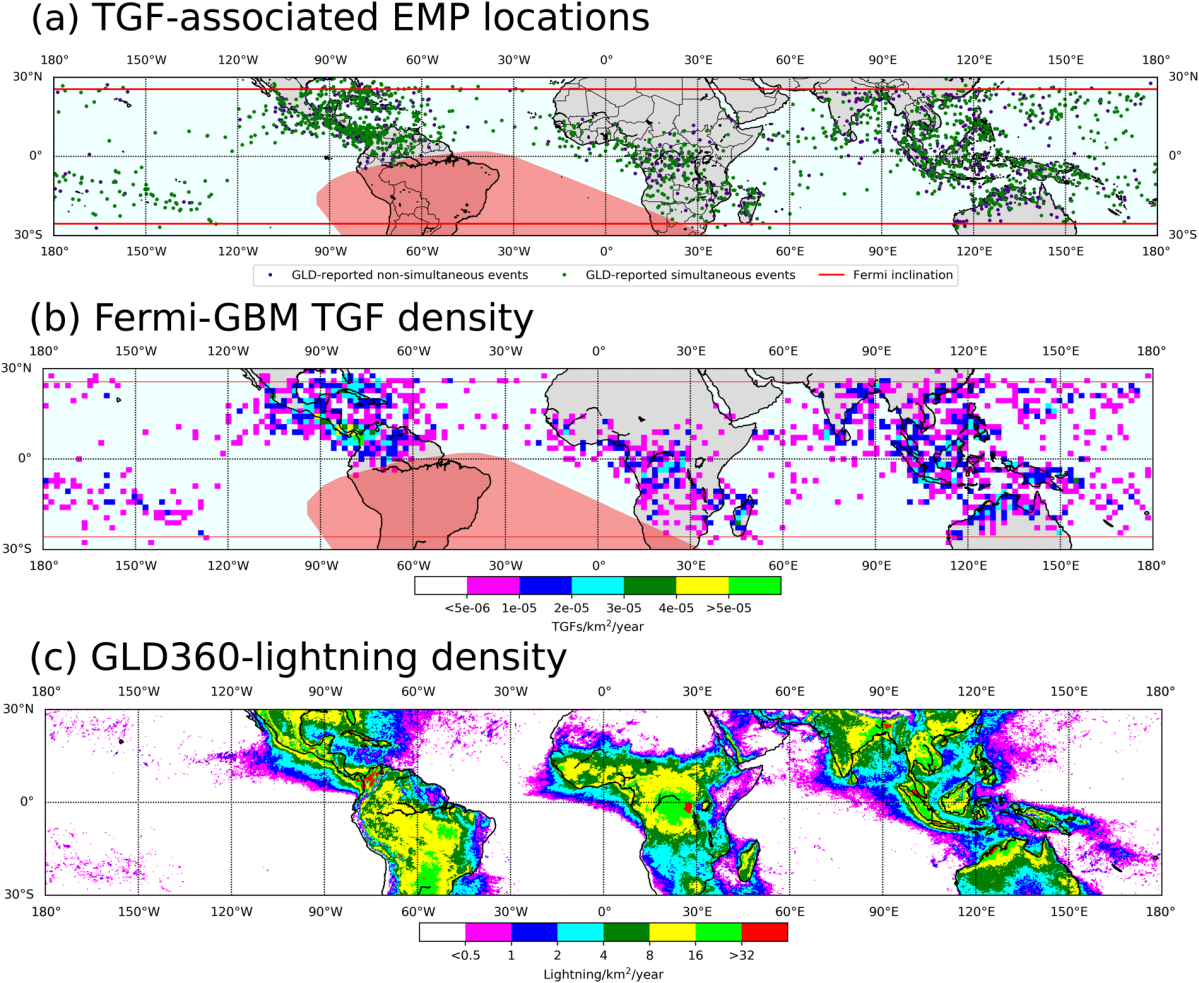
(**a**) Map showing locations of 2266 GLD360-reported EMPs that occurred within 3.5 ms of 2188 TGFs reported by the Fermi-GBM in 2013–2017. Green dots (GLD360-reported simultaneous events) indicate the locations of 1545 EMPs occurring within 200 μs of 1502 TGFs. Purple dots (GLD360-reported non-simultaneous events) indicate the locations of 721 EMPs occurring in the 200 μs–3.5 ms time window before or after the other 686 TGFs. The red lines indicate the 25.6°N orbital inclination of Fermi. Note that some dots overlap with each other due to the spatial proximity of their locations. (**b**) Map showing the TGF density (TGFs/km^2^/year, computed using 20 km × 20 km grid-cells) for the 2188 TGFs from (**a**), and (**c**) Map showing the GLD360-reported lightning flash density (lightning flashes/km^2^/year, computed using 0.25° × 0.25° grid-cells) for the same 5-year period as for the TGFs. Lightning flash density is many orders of magnitude higher than the TGF density reported by the Fermi-GBM, allowing the use of finer grid-cells for plotting the former. Generally speaking, regions with relatively high TGF densities coincide with those having relatively high lightning densities, although regional differences in the TGF/lightning ratio have been reported (see, for example, Mailyan *et al*.^25^ and references therein). Standard Python 2.7 basemap from Anaconda distribution (https://www.anaconda.com/distribution/) was used to produce the plots.

In this paper, we examine the characteristics of the two categories of TGFs: those for which associated EMPs were reported to occur simultaneously (within ±200 μs of a TGF’s start-time) and those for which associated EMPs were reported to occur non-simultaneously (in the 200 μs–3.5 ms time-window before or after TGFs). See also the Methods section for further discussion. EMPs and TGFs were reported, respectively, by a ground-based LLS (Global Lightning Dataset or GLD360) and a satellite-based detector (Fermi Gamma Ray Burst Monitor or Fermi-GBM). See the Methods section for details on how EMPs and TGFs were correlated. We examined an unprecedentedly large dataset of 3860 TGFs reported by the Fermi-GBM during five years (2013–2017) over its entire coverage region (see Fig. 1). 2188 (57%) of these TGFs were accompanied, within 3.5 ms of their start-time, by GLD360-reported EMPs. 74 (3.4%) of these 2188 TGFs were each accompanied by two EMPs within 3.5 ms, and two (0.09%) were each accompanied by three EMPs. For 1502 (69%) of the 2188 TGFs, the EMPs occurred simultaneously. Of these 1502 TGFs, 34 (2.3%) were accompanied by two simultaneous EMPs within 200 μs of the TGF. For 686 (31%) of the 2188 TGFs, the associated EMPs were reported to occur non-simultaneously.

### Peak amplitudes of TGF-associated EMPs reported by GLD360

**Figure 2:**
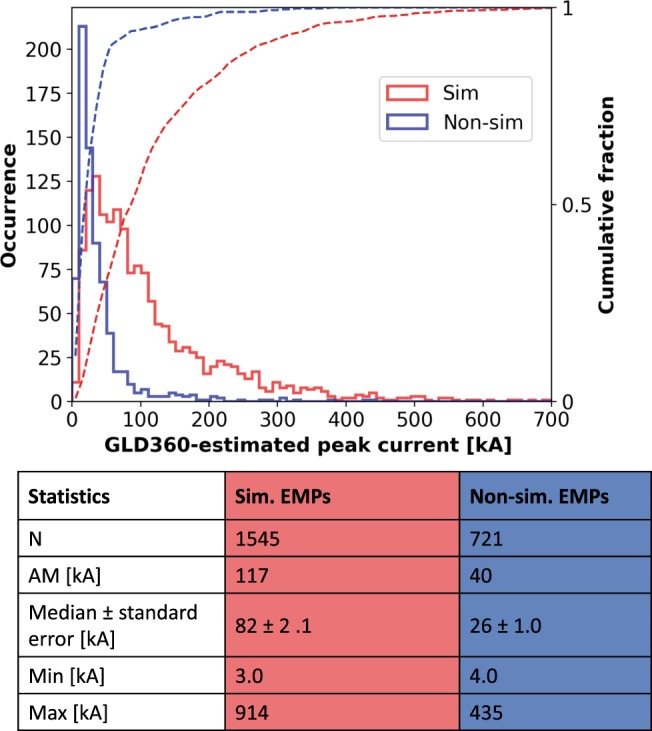
Histogram of GLD360-estimated peak currents (I_p_) for EMPs occurring simultaneously (light-red outlined bars) and non-simultaneously (light-blue outlined bars) with TGFs reported by the Fermi-GBM in 2013–2017. Sample size (N), arithmentic mean (AM), median (±standard error), minimum (Min) and maximum (Max) values are shown for each category of EMPs. The standard errors were computed by dividing the median value by the square root of the sample size. The “bootstrap method” used by Briggs *et al*.^29^, gives comparable results for the standard errors. The horizontal axis is truncated at 700 kA; only 2 EMPs had higher peak currents.

Figure 2 shows the histogram of the GLD360-estimated peak currents for EMPs that were simultaneous (light-red outlined bars) and non-simultaneous (light-blue outlined bars) with TGFs. For the 1545 EMPs that occurred simultaneously with 1502 TGFs, the peak currents ranged from 3 to 914 kA, with the median (±standard error) being 82 (±2.1) kA. 1094 (71%) of these EMPs had peak currents greater than 50 kA and 387 (25%) had peak currents greater than 150 kA. For comparison, the median peak current for first strokes in negative cloud-to-ground (CG) lightning (which comprise 90% of all CG lightning and for which “ground-truth” current measurements are available) is 30 kA (Rakov and Uman^28^, Ch. 1). The peak currents for 721 TGF-associated non-simultaneous EMPs ranged from 4 to 435 kA, with the median being 26 (±1) kA. As discussed in the Methods section, the GLD360-estimated peak current should be viewed as a quantity proportional to the distance-normalized peak-amplitude of the magnetic radiation field of EMPs, rather than the peak current of their sources. Therefore, the median peak-amplitude of TGF-associated EMPs is 3.2 times higher for simultaneous versus non-simultaneous EMPs.

### Relationship between TGF-EMP peak amplitudes and TGF-source characteristics

**Figure 3:**
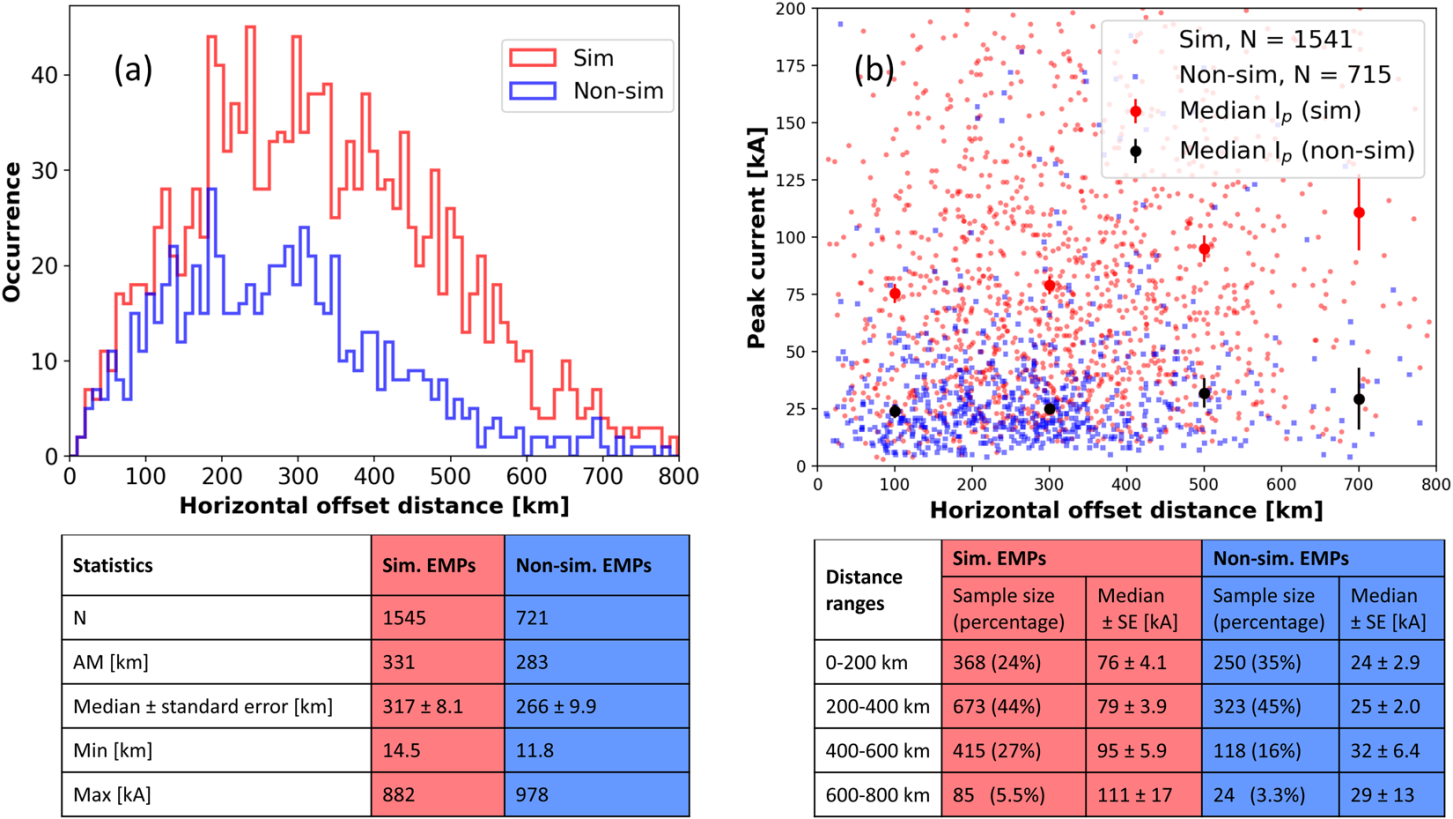
(**a**) Histogram of horizontal offset distances between the Fermi spacecraft-nadir and GLD360-located EMPs occurring simultaneously (light-red outlined bars) and non-simultaneously (light-blue outlined bars) with TGFs reported by Fermi-GBM in 2013–2017. Sample size (N), arithmentic mean (AM), median (±standard error), minimum (Min) and maximum (Max) values are shown for each category of EMPs. (**b**) Scatter plot showing the GLD360-estimated peak currents for EMPs occurring simultaneously (light red dots) and non-simultaneously (light blue dots) with TGFs versus their horizontal offset distances. The median peak current for 200 km distance-bins are shown using red and black dots for simultaneous and non-simultaneous EMPs, respectively, and the standard errors are represented by red and black vertical lines, respectively. The numbers and percentages of each type of EMPs in different distance ranges along with the respective median (±standard error (SE)) peak currents are provided in the table in (**b**). The standard errors were computed by dividing the median value for a sample by the square root of the sample size. The vertical (peak current) axis was truncated at 200 kA to clearly demonstate the variation in the median peak currents at different distance ranges. These medians are also shown in the table below the scatter plot. In both (**a**,**b**), the horizontal axes are truncated at 800 km; only 10 EMPs occurred at farther offset distances. These 10 EMPs are not included in the statistics in (**b**).

We examined the dependence of the GLD360-estimated TGF-associated EMP-peak-current on the horizontal offset distance between the Fermi spacecraft-nadir and the TGF source-location determined by GLD360. From Fig. 3a we see that the median (±standard error) offset distances were 317 (±8.1) and 266 (±9.9) km for TGFs with and without simultaneous EMPs, respectively. TGFs are detected by the Fermi-GBM when their energies exceed the instrumentation detection-threshold. TGFs with sources at farther horizontal distances from the spacecraft suffer greater attenuation due to propagation in the Earth’s atmosphere before being detected by the GBM, so such TGFs are expected be intrinsically brighter (more intense). Therefore, the offset distance being significantly longer for TGFs with simultaneous EMPs is an indication that these TGFs produce more intense gamma-ray signatures detectable from larger distances. From Fig. 3b we see that the median peak current for EMPs at offset distances of 600–800 km is 111 kA, which is significantly higher (standard errors are much smaller than the difference between the medians), than 76 kA for EMPs at offset distances of 0–200 km. On the other hand, the median peak current for TGFs without simultaneous EMPs are similar (standard errors are comparable to or larger than the difference between the medians), 24 and 29 kA, respectively, at those distance ranges. Thus, TGFs with simultaneous EMPs that occur at farther offset distances have both a higher RF peak amplitude and a brighter gamma ray signature. This inference drawn from the analysis of two independent measurements (TGF detected from space and EMPs detected from ground) provides the evidence that for TGFs with simultaneous EMPs, the EMPs and TGFs are produced by the same phenomenon, rather than the EMPs being from “regular” lightning in TGF-producing thunderstorms.

Nag *et al*.^30^ show using observations and modeling that for relatively compact (small spatial extent) cloud lightning flashes with channel lengths on the order of 100 m occurring 15 km above the Earth’s surface, the RF bipolar radiation EMP measured at ground is produced by a unipolar source current traveling over the relatively short channel length. Additionally, they show that since LLSs assume that the peak current is proportional to the peak field (which is accurate for cloud-to-ground lightning), they tend to significantly underestimate the peak current of compact intracloud discharges (CIDs), for which the peak of the RF radiation field is proportional to the peak of the time-derivative of the source current (dI/dt). If we assume that TGFs have a vertical extent of a few hundred meters and if runaway electrons and the resulting ionization are directly generating the TGF source current, then we can view the TGF EMP measured by LLS-sensors at distances of many tens to hundreds of kilometers as the radiation field due to this TGF source current. In such a scenario, the peak amplitude of this EMP would be proportional to dI/dt and the LLS-estimated peak current would be an underestimate of the real TGF source peak current. If the above assumptions about TGF phenomenology are correct, the median peak current of 82 kA for TGFs with simultaneous EMPs reported by GLD360 discussed in the previous section should be treated as an underestimate. Also, non-simultaneous EMPs could be viewed as lightning-produced pulses in TGF-producing thunderstorms which were reported by GLD360, rather than signatures of the TGFs themselves. The absence of a simultaneous EMP accompanying a TGF could be due to a significantly-non-vertical propagation path of the TGF source current. This would result in the production of a TGF-EMP radiation field signature that would not be suitable for detection by multiple GLD360 sensors (as is needed for EMP-geolocation) which measure changes in the vertical component of electric and horizontal (azimuthal) component of magnetic field.

### Characteristics of energetic emmissions reported by the Fermi GBM for the two categories of TGFs

For 1489 out of 1502 TGFs with simultaneous EMPs reported by GLD360, TGF-durations, hardness ratios, and photon counts could be estimated (see Methods section). Also, these parameters could be estimated for 672 out of 686 TGFs with non-simultaneous EMPs.

**Figure 4:**
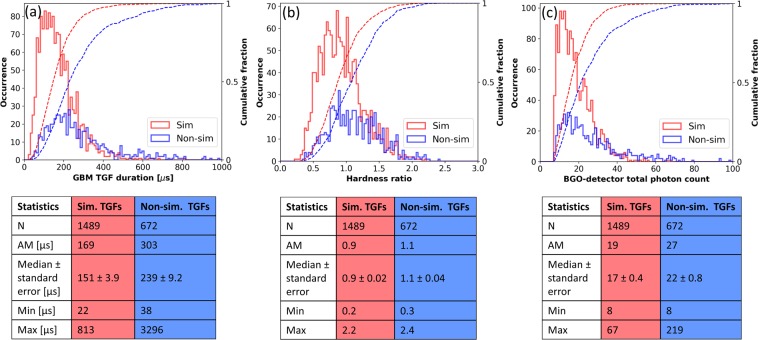
Histograms of (**a**) duration (**b**) hardness ratio and (**c**) total photon count for TGFs reported by Fermi-GBM in 2013–2017 with accompanying simultaneous (light-red outlined bars) and non-simultaneous (light-blue outlined bars) EMPs. Also shown are the cumulative distributions of these parameters for the two types of TGFs using red and blue lines, respectively. Sample size (N), arithmentic mean (AM), median (±standard error), minimum (Min) and maximum (Max) values are shown for each category of TGFs. The standard errors were computed by dividing the median value for a sample by the square root of the sample size.

From Fig. 4a, we see that for TGFs with simultaneous EMPs, the durations ranged from 22 to 813 μs, with the median (±standard error) being 151 (±3.9) μs. For TGFs with non-simultaneous EMPs for which durations could be estimated, the durations ranged from 38 μs to 3.3 ms, with the median being 239 (±9.2) μs. 70% and 39% of the TGFs with accompanying simultaneous and non-simultaneous EMPs, respectively, had durations less than 200 μs. Clearly, TGFs with accompanying simultaneous EMPs tend to have significantly shorter durations, with their median duration being 1.6 times shorter than that for TGFs with non-simultaneous EMPs. For comparison, the median duration for 3774 TGFs reported by Fermi-GBM in 2013–2017 (for which durations could be estimated), regardless of whether they were accompanied by EMPs or not, was 191 μs. Since TGFs with simultaneous EMPs also have significantly larger GLD360-reported peak currents, our results therefore indicate that such TGFs have both a shorter median duration and a higher median peak amplitude.

For TGFs with simultaneous EMPs, we see from Fig. 4b that the hardness ratios ranged from 0.2 to 2.2, with the median (±standard error) being 0.9 (±0.02). Hardness ratios for TGFs with non-simultaneous EMPs ranged from 0.3 to 2.4 with the median being 1.1 (±0.04). 63 and 44% of the TGFs with accompanying simultaneous and non-simultaneous EMPs, respectively, had hardness ratios less than 1. A hardness ratio of 1 for a TGF indicates that the Fermi-GBM reported an equal number of photon-counts with energies greater than and less than 300 keV. Therefore, it is apparent that TGFs with accompanying simultaneous EMPs have a median hardness ratio that is significantly smaller than that for TGFs with non-simultaneous EMPs. For comparison, the median hardness ratios for 3821 TGFs reported by Fermi-GBM in 2013–2017 (for which hardness ratios were available), regardless of whether they were accompanied by EMPs or not, was 0.98.

Figure 4c shows that for TGFs with simultaneous EMPs, the total photon counts ranged from 8 to 67, with the median (±standard error) being 17 (±0.4). For TGFs with non-simultaneous EMPs the total photon counts ranged from 8 to 219, with the median being 22 (±0.8). 2 and 16% of the TGFs with accompanying simultaneous and non-simultaneous EMPs, respectively, had total photon counts greater than 40. So, TGFs with accompanying simultaneous EMPs have a significantly smaller median total-photon-count reported by the Fermi GBM than that for TGFs with non-simultaneous EMPs. For comparison, the median BGO-detector total-photon-count for 3821 TGFs reported in 2013–2017 by the Fermi-GBM (for which photon counts were available), regardless of whether they were accompanied by EMPs or not, was 19.

In summary, from the above analysis we conclude that TGFs with accompanying simultaneous EMPs tend to have shorter durations, smaller number of photons with energies greater than 300 keV reported by the Fermi-GBM (smaller hardness ratios), and lower total photon counts. The impact on these parameters from instrumental and other effects (pulse pile-up, dead-time, Compton scattering, and atmospheric attenuation) are discussed in the Methods section.

## Methods

### Global lightning dataset (GLD360)

The Global Lightning Dataset (GLD360) is obtained from a global LLS which has been in operation since September 2009, with data being made available to users since May 2011. The GLD360 employs magnetic field sensors sensitive to the VLF range, located around the world. EMPs measured by multiple sensors are geolocated using a combination of time-of-arrival and magnetic-direction-finding methods in conjunction with a lightning waveform feature recognition algorithm^31,32^. Additionally, electromagnetic wave travel-time corrections and a propagation model are applied to fine-tune the geolocation, and the measured magnetic field peak-amplitudes of EMPs. The peak currents of EMP-sources are estimated from their magnetic field peaks, after distance-normalization (to 100 km) and compensation for propagation effects on the electromagnetic waves in the Earth-ionosphere waveguide, using an empirical field-to-current-conversion equation appropriate for negative cloud-to-ground lightning subsequent return strokes^30,32^. The median location error and peak current estimation error for GLD360 are 2 km and 27%, respectively^33^. For a TGF-associated EMP, the GLD360-estimated peak current can be viewed as a quantity proportional to the distance-normalized (to 100 km) peak-amplitude of the magnetic radiation field.

### Fermi gamma ray burst monitor (GBM)

The GBM is an auxiliary instrument onboard the Fermi Gamma-ray Space Telescope comprising of 12 sodium iodide (NaI) and two bismuth germanate (BGO) detectors^34^. Particles with effective energy ranges of 8–1000 keV and 0.2–40 MeV are measured by the NaI and BGO detectors, respectively. The timing precision of the measurements is 2 μs and the absolute accuracy is several microseconds. The Fermi-GBM is capable of measuring TGFs within a horizontal distance of up to about 800 km from the spacecraft’s footprint^35^. After implementation of new ground-search algorithms in 2012, the TGF detection rate improved to about 800 events per year^29^. The Fermi-GBM TGF catalog provides the photon counts per 2 μs for each of the two BGO detectors, cumulative photon counts for the 12 NaI detectors per 2 μs (10 μs if the NaI detectors are saturated), spacecraft position, TGF start-time measured at the spacecraft altitude, and the duration of the discovery-bin. The discovery-bin is the time-window in the ground-search algorithm corresponding to the most significant joint Poisson probability of occurrence of an identified TGF^29,35^. The discovery-bin start-time is a reasonable approximation for the TGF photon flux start-time^24^.

**Figure 5:**
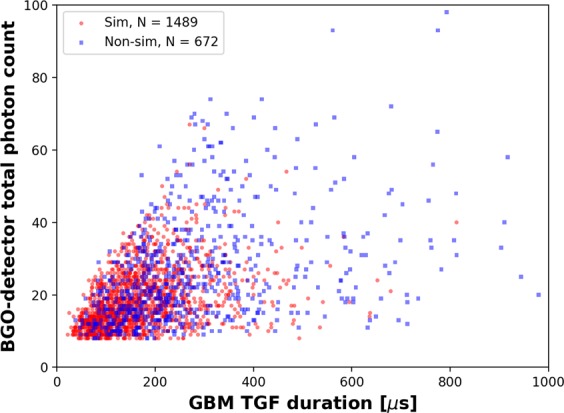
Scatter plot showing the GBM-reported BGO-detector total photon counts for TGFs with EMPs occurring simultaneously (light red dots) and non-simultaneously (light blue squares) versus TGF durations.

The TGF-duration shown in Fig. 4a was estimated using the BGO-detector data and the Bayesian Block (BB) algorithm^35,36^. The BB algorithm identifies statistically significant events by dividing the data into piecewise blocks of time and finding the optimal boundaries for each time-block using non-parametric analysis methods. The total photon counts shown in Fig. 4c are those reported by the BGO detectors within the TGF time-windows found using the BB algorithm. Note that the BGO-detector temporal resolution of 2 μs and dead-time of 2.6 μs are more than and nearly more than an order of magnitude shorter, respectively, than the shortest TGF duration of 22 μs reported in Fig. 4a. The median duration for TGFs with simultaneous EMPs is nearly two orders of magnitude longer than the temporal resolution and dead-time. However, the instrumental dead-time along with detector-sensitivity limitations likely result in the GBM not observing any short duration TGFs with high total photon counts^29^. This effect is seen in Fig. 5 which shows the scatter plot of TGF total-photon-counts versus their durations. These instrumental limits could, therefore, cause the GBM-reported durations for the shortest TGFs to be overestimated.

The hardness ratio shown in Fig. 4b was computed for each GBM-reported TGF for which the BB-algorithm-estimated duration was available. It is defined as the ratio of photon-counts reported by Fermi’s NaI detectors for photons with energies greater than and less than 300 keV. So, TGFs with larger hardness ratios (larger fraction of photons with energies greater than 300 keV), correspond to TGFs with harder spectra. The 2 μs (10 μs if NaI detectors are saturated) temporal resolution of the NaI detectors will result in pulse pile-up^37,38^, which occurs when two or more lower energy photons arrive at the detectors within 2 μs (or 10 μs) of each other and are reported as a single higher energy photon. For short-duration TGFs with high photon-counts this will lead to higher values of hardness ratios, since the GBM could overestimate the counts of higher energy photons due to pulse-pile up. Pulse-pile up would also cause the BGO-detector-reported photon counts to be underestimated for shorter duration TGFs. Since the median duration of TGFs with simultaneous EMPs is significantly shorter than that for TGFs without simultaneous EMPs, pulse pile-up can therefore result in overestimation of the hardness ratios and underestimation of the total photon counts more often for the former class of TGFs than for the latter.

Compton scattering can result in increased counts of lower energy photons as well as increased durations for TGFs occurring at farther distances from the detectors. Thus, for such TGFs, the hardness ratios would be underestimated. Compton scattering is expected to more significantly impact GBM NaI-detector measurements (with an energy-range lower-limit of about 8 keV) than those from the BGO detectors (with an energy-range lower limit of about 200 keV). The effect of Compton scattering on TGF spectral and temporal characteristics have been explored in detail in previous studies^5,38–41^.

**Figure 6:**
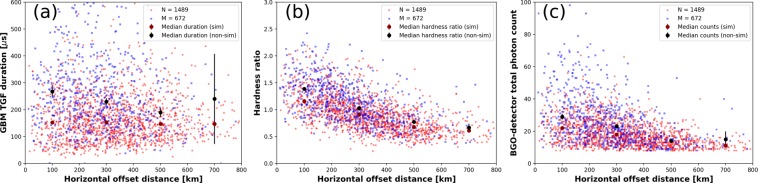
Scatter plot showing the (**a**) durations, (**b**) hardness ratios, and (**c**) total photon counts for TGFs with EMPs occurring simultaneously (light red dots) and non-simultaneously (light blue dots) versus their horizontal offset distances from the spacecraft. The median values of each parameter for 200 km distance-bins are shown using dark-red and black dots for simultaneous and non-simultaneous EMPs, respectively, and the respective standard errors are represented by dark-red and black vertical lines, respectively.

In our dataset, we found that, generally speaking, the median TGF duration, hardness ratio, and total photon count were all lower for TGFs with simultaneous EMPs than for those without such EMPs at the same horizontal distance ranges (see Fig. 6a–c). The only exceptions were for the TGF durations at the 600–800 km range for which a statistically significant difference in the median values could not be determined and for the total photon counts at the 400–600 km range for which the median values were similar for the two classes of TGFs. Additionally, for both classes of TGFs, all three parameters showed a general decrease with increasing distance, even though this decrease is less pronounced for the median duration of TGFs with simultaneous EMPs. TGF photons propagating over larger distances will be more affected by Compton scattering and atmospheric attenuation, and the variation with distance in the median values of the three Fermi GBM-observed parameters can very likely be attributed to these effects.

It remains to be seen, what the relative contributions to the observed differences in the Fermi GBM-derived characteristics of the two classes of TGFs (see Fig. 4) are from instrumental effects versus from differences in their source-characteristics.

### TGF-EMP correlation

To identify TGF-associated EMPs, we searched for GLD360-geolocated EMPs occurring within a ± 6.5-ms time-window around a GBM-reported TGF’s start-time. We assumed that the two-dimensional TGF source location was the same as that of the EMP that was closest in time to the TGF. Then, light travel-time correction was performed^24^. The ±6.5-ms time-window was selected to ensure that after light travel-time correction was performed, any EMPs occurring within (the smaller time-window of) ±3.5 ms of TGFs were not missed. EMPs were defined to occur simultaneously with a TGF if they occurred within ±200 μs of the TGF’s start-time. This definition has been used in previous GBM-TGF studies^21^. EMPs occurring outside this time-window were defined to be non-simultaneous with the respective TGF.

**Figure 7:**
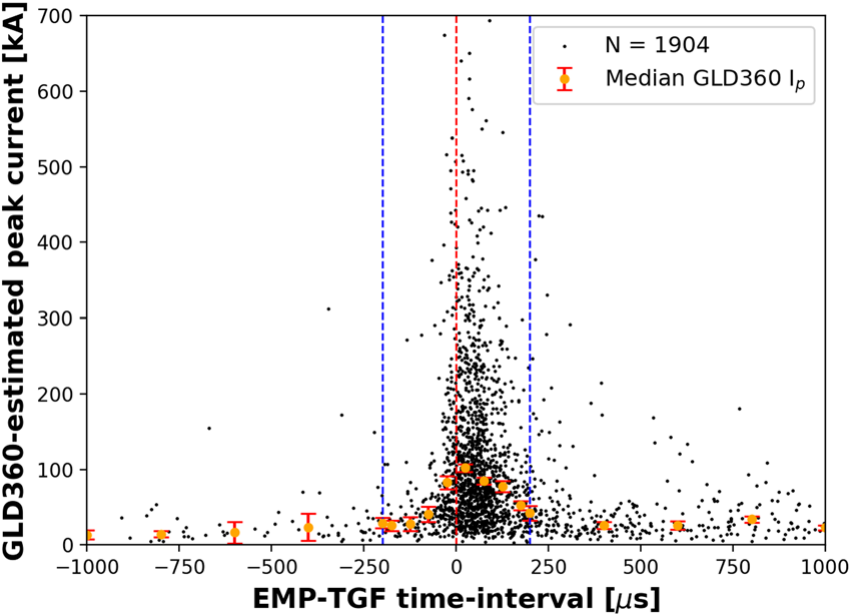
Scatter plot (black dots) showing the GLD360-estimated peak currents for EMPs versus the EMP-start-to-TGF-start time-intervals. The horizontal (time) and vertical (peak current) axes were truncated at ±1000 μs and 700 kA, respectively; 1904 (out of 2266) EMPs occurred within these limits. The red vertical dashed line indicates the position on the time-axis at which the EMP-TGF time-interval is zero. The blue vertical dashed lines indicate the ±200-μs time window within which EMPs were consdiered to be simultaneous with TGFs. The orange dots indicate the median GLD360-reported peak currents over 50-μs bins for simultaneous EMPs and over 200-μs bins for non-simultaneous EMPs. The vertical bar at each median value indicates the standard error (standard deviation divided by the square root of sample size in each bin). We used a larger bin-size outside the ±200 μs time-window in order to avoid bins with very small sample sizes and obtain more robust median values with reasonable standard errors.

We examined the time intervals between the GLD360-reported pulse start-times and the GBM-reported TGF start-times for 2266 EMPs associated with 2188 TGFs. As can be seen from Fig. 7, a large majority (1819 of 2266 or 80%) of EMPs started after the respective gamma-ray flux’s start-time. For these EMPs, the absolute values of the EMP-TGF time-intervals ranged from 1 μs to 3.5 ms, with the median being 91 μs. The start-times of 447 (20%) of 2266 EMPs preceded the respective TGF’s GBM-reported start-time. The time-intervals for these EMPs ranged from 1 μs to 3.4 ms, with the median being 65 μs. Note that, the expected error in the time parameters is on the order of 10 μs due to uncertainties in TGF source-altitude and two-dimensional location^24^. For 1545 simultaneous EMPs accompanying 1502 TGFs, the median absolute EMP-TGF time-interval was 55 μs. This is similar to the 50 μs EMP-TGF time interval reported by Mailyan *et al*.^24^ for TGF-simultaneous EMPs geolocated by the U.S. National Lightning Detection Network. 1244 (81%) of the 1545 simultaneous EMPs started after the TGF start-time. The median peak currents (orange dots in Fig. 7) were higher for EMPs whose start-times were closer to TGF-start-times. Additionally, the median peak current for simultaneous EMPs whose start-times occurred after the GBM-reported TGF start-time (in the 0 to +200 μs time window in Fig. 7) was 86 kA which is somewhat higher than the median peak current of 60 kA for EMPs whose start-times occurred before the TGF start-time (in the −200 to 0 μs time window in Fig. 7).

## References

[1] Fishman GJ (1994). Discovery of intense gamma-ray flashes of atmospheric origin. Science.

[2] Carlson BE, Lehtinen NG, Inan US (2009). Terrestrial gamma ray flash production by lightning current pulses. Journal of Geophysical Research: Space Physics.

[3] Celestin S, Pasko VP (2012). Compton scattering effects on the duration of terrestrial gamma-ray flashes. Geophysical Research Letters.

[4] Dwyer JR (2012). The relativistic feedback discharge model of terrestrial gamma ray flashes. J. Geophys. Res..

[5] Moss G, Pasko VP, Liu N, Veronis G (2006). Monte Carlo model for analysis of thermal runaway electrons in streamer tips in transient luminous events and streamer zones of lightning leaders. J. Geophys. Res..

[6] Dwyer JR (2003). A fundamental limit on electric fields in air. Geophys. Res. Lett..

[7] Gurevich AV, Milikh GM, Rouseel-Dupre R (1992). Runaway electron mechanism of air breakdown and preconditioning during a thunderstorm. Physics Letters A.

[8] Briggs MS (2010). First results on terrestrial gamma ray flashes from the Fermi Gamma-ray Burst Monitor. Journal of Geophysical Research: Space Physics.

[9] Dwyer JR (2004). A ground level gamma-ray burst observed in association with rocket-triggered lightning. Geophys. Res. Lett..

[10] Dwyer JR (2012). Observation of a gamma-ray flash at ground level in association with a cloud-to-ground lightning return stroke. J. Geophys. Res..

[11] Tran MD (2015). A terrestrial gamma-ray flash recorded at the Lightning Observatory in Gainesville, Florida. Journal of Atmospheric and Solar-Terrestrial Physics.

[12] Hare BM (2016). Ground-level observation of a terrestrial gamma ray flash initiated by a triggered lightning. J. Geophys. Res. Atmos..

[13] Abbasi RU (2018). Gamma ray showers observed at ground level in coincidence with downward lightning leaders. Journal of Geophysical Research: Atmospheres.

[14] Wada Y (2019). Gamma-ray glow preceding downward terrestrial gamma-ray flash. Communications Physics.

[15] Smith DM (2011). A terrestrial gamma ray flash observed from an aircraft. J. Geophys. Res..

[16] Bowers GS (2018). A Terrestrial Gamma-Ray Flash inside the Eyewall of Hurricane Patricia. Journal of Geophysical Research: Atmospheres.

[17] Dwyer JR, Cummer SA (2013). Radio emissions from terrestrial gamma-ray flashes. Journal of Geophysical Research: Space Physics.

[18] Cummer SA (2011). The lightning-TGF relationship on microsecond timescales. Geophys. Res. Lett..

[19] Lyu F, Cummer SA, McTague L (2015). Insights into high peak current in-cloud lightning events during thunderstorms. Geophys. Res. Lett..

[20] Connaughton V (2010). Associations between Fermi Gamma-ray Burst Monitor terrestrial gamma ray flashes and sferics from the World Wide Lightning Location Network. Journal of Geophysical Research: Space Physics.

[21] Connaughton V (2013). Radio signals from electron beams in terrestrial gamma-ray flashes. J. Geophys. Res. Space Physics.

[22] Mezentsev A (2016). Radio emissions from double RHESSI TGFs. Journal of Geophysical Research: Atmospheres.

[23] Lyu FS (2018). Very high frequency radio emissions associated with the production of terrestrial gamma-ray flashes. Geophysical Research Letters.

[24] Mailyan BG (2018). Characteristics of Radio Emissions Associated with Terrestrial Gamma-Ray Flashes. Journal of Geophysical Research: Space Physics.

[25] Mailyan, B. G. *et al*. Characteristics of Lightning Associated with Terrestrial Gamma-ray Flashes in Various Geographical Regions. Proceedings of 25th International Lightning Detection Conference March 12-15, Fort Lauderdale, Florida, USA (2018b).

[26] Mailyan, B. G. *et al*. Investigating Radio Emissions Associated with Terrestrial Gamma-ray Flashes using NLDN, GLD360 and Fermi-GBM. Proceedings of XVI International Conference on Atmospheric Electricity 17 -22 June 2018, Nara city, Nara, Japan (2018c).

[27] Nag A, Murphy MJ, Schulz W, Cummins KL (2015). Lightning locating systems: Insights on characteristics and validation techniques. Earth and Space Science.

[28] Rakov, V. A. & Uman, M. A. Lightning: physics and effects. Cambridge University Press (2003).

[29] Briggs MS (2013). Terrestrial gamma-ray flashes in the Fermi era: Improved observations and analysis methods. Journal of Geophysical Research: Space Physics.

[30] Nag A, Rakov V, Cramer J (2011). Remote measurements of currents in cloud lightning discharges. IEEE Transactions on Electromagnetic Compatibility.

[31] Said, R. K., Murphy, M. J., Demetriades, N., Inan, U. S. & Cummins, K. L. Initial performance estimates of the GLD360 lightning detection network. Abstract #AE33A-0255 presented at 2010 Fall Meeting, AGU, San Francisco, Calif, (2010).

[32] Said RK (2017). Towards a global lightning locating system. Weather.

[33] Mallick, S. *et al*. Evaluation of the WWLLN performance characteristics using rocket-triggered lightning data. In Intl. Conf. on Grounding and Earthing/6th Intl. Conf. on Lightning Physics and Effects (2014).

[34] Meegan C (2009). The Fermi Gamma-ray Burst Monitor. The Astrophysical Journal.

[35] Roberts OJ (2018). The first Fermi-GBM terrestrial gamma ray flash catalog. Journal of Geophysical Research: Space Physics.

[36] Scargle JD, Norris JP, Jackson B, Chiang J (2013). Studies in astronomical time series analysis. VI. Bayesian block representations. The Astrophysical Journal.

[37] Chaplin V, Bhat N, Briggs MS, Connaughton V (2013). Analytical modeling of pulse-pileup distortion using the true pulse shape; applications to Fermi-GBM. Nuclear Instruments and Methods in Physics Research Section A: Accelerators, Spectrometers, Detectors and Associated Equipment.

[38] Mailyan BG (2016). The spectroscopy of individual terrestrial gamma-ray flashes: Constraining the source properties. J. Geophys. Res. Space Physics.

[39] Grefenstette BW, Smith DM, Dwyer JR, Fishman GJ (2008). Time evolution of terrestrial gamma ray flashes. Geophysical Research Letters.

[40] Østgaard N, Gjesteland T, Stadsnes J, Connell PH, Carlson B (2008). Production altitude and time delays of the terrestrial gamma flashes: Revisiting the Burst and Transient Source Experiment spectra. J. Geophys. Res..

[41] Fitzpatrick G (2014). Compton scattering in terrestrial gamma-ray flashes detected with the Fermi Gamma-ray Burst Monitor. Phys. Rev. D.

